# Experimental study and thermodynamic modeling of the Al–Co–Cr–Ni system

**DOI:** 10.1088/1468-6996/16/5/055001

**Published:** 2015-09-21

**Authors:** Thomas Gheno, Xuan L Liu, Greta Lindwall, Zi-Kui Liu, Brian Gleeson

**Affiliations:** 1 Department of Mechanical Engineering and Materials Science, University of Pittsburgh, Pittsburgh, PA 15261, USA; 2 Department of Materials Science and Engineering, The Pennsylvania State University, University Park, PA 16802, USA

**Keywords:** bondcoat, phase compositions, phase equilibrium, Calphad, MCrAlY

## Abstract

A thermodynamic database for the Al–Co–Cr–Ni system is built via the Calphad method by extrapolating re-assessed ternary subsystems. A minimum number of quaternary parameters are included, which are optimized using experimental phase equilibrium data obtained by electron probe micro-analysis and x-ray diffraction analysis of NiCoCrAlY alloys spanning a wide compositional range, after annealing at 900 °C, 1100 °C and 1200 °C, and water quenching. These temperatures are relevant to oxidation and corrosion resistant MCrAlY coatings, where M corresponds to some combination of nickel and cobalt. Comparisons of calculated and measured phase compositions show excellent agreement for the *β*–*γ* equilibrium, and good agreement for three-phase *β*–*γ*–*σ* and *β*–*γ*–*α* equilibria. An extensive comparison with existing Ni-base databases (TCNI6, TTNI8, NIST) is presented in terms of phase compositions.

## 1. Introduction

Nickel- and cobalt-base alloys are widely used in high temperature structural applications, such as aero, marine and land-based gas turbines. Coherent precipitation of geometrically close-packed *γ*′ (L1_2_, ordered cubic based on Ni_3_Al) in the *γ* (A1, fcc solid solution based on Ni) matrix provides outstanding high temperature strength to Ni-base superalloys, which are found in high pressure turbine disks and blades, where the thermo-mechanical load is most demanding [1, 2]. However, the continuous increase in operating temperatures and in the chemically aggressive character of combustion environments has prompted the development of oxidation and corrosion resistant coatings [3–5]. Two main types of materials are currently in use: diffusion coatings based on *β*-NiAl (B2, ordered cubic), and overlay MCrAlY (M = Ni, Co or both) coatings based on a *β*–*γ* microstructure.

Conventional Co-base superalloys rely on carbide and solid-solution strengthening and have been confined to moderately high temperature components (700 °C–900 °C) such as turbine vanes [6]. The recent discovery of a stable *γ*′ Co_3_(Al, W) compound [7] has inspired the development of novel high strength Co-base alloys [8–12]. Their high melting point makes them promising candidates for higher service temperatures and, ultimately, increased efficiencies. However, their poor oxidation resistance [13, 14] requires the use of coatings, and MCrAlYs are considered for this purpose [15].

As modern MCrAlYs often contain significant amounts of both nickel and cobalt, a sound description of the Al–Co–Cr–Ni system’s thermodynamic properties is desired to ensure an optimal design of these coatings and control of their microstructural evolutions. Yet, while thermodynamic models and databases exist which cover this quaternary system in principle, evaluations against experimental data are scarce, mostly qualitative and partial at best; no thorough assessment has been openly reported.

Common MCrAlY coatings have a primary *β*–*γ* microstructure at high temperatures, above about 1100 °C. At temperatures typical of service conditions (800 °C–1000 °C), the coatings may also form *γ*′, *σ* or *α*, depending on their specific composition (*σ*: Frank–Kasper based on CoCr; *α*: A2, bcc solid solution based on Cr). Achar *et al* [16] used the TTNI database from Thermotech (version not specified) to calculate phase equilibria for a large number of NiCoCrAl alloys in the temperature range 950 °C–1050 °C. However, the comparison with experimental data was limited to the identification of the phases formed, and phase compositions were not reported. The authors concluded on a good agreement between experimental and predicted phase constitutions, except for cobalt contents in excess of 20 wt.%. Similarly, Ma and Schoenung [17] reviewed published reports of experimental phase constitutions and discussed phase equilibria in the quaternary system predicted by the TTNI7 database, but they did not evaluate the database against experimental data quantitatively. Brož *et al* [18, 19] reported experimental phase compositions together with phase equilibria calculated using a developmental database, with a focus on the *γ*–*γ*′ equilibrium. However, they did not publish the details of their thermodynamic modeling.

Several multi-component phase equilibrium calculations including Al, Co, Cr and Ni have been recently published in relation with the development of ‘high entropy alloys’ [20] based on AlCoCrCuFeNi compositions. Again these studies (see for example [21, 22]) report qualitative comparisons of experimental versus calculated phase constitutions using commercial databases, which does not allow these databases to be evaluated with a satisfactory accuracy. Furthermore, the thermodynamic models and parameters underlying these databases are not openly available, and cannot therefore be examined.

The present paper reports a new, developmental Al–Co–Cr–Ni database built by the Calphad method from re-assessed ternary subsystems [23]. The database is provided in a non-encrypted format. A quantitative evaluation of this and existing databases is provided, based on new experimental data obtained from NiCoCrAlY alloys in the temperature range 900 °C–1200 °C. A variety of compositions were used to cover a large part of the quaternary system. In the future, our database will be updated to include reactive elements such as yttrium or hafnium.

## 2. Experimental procedures

Ingots of nominal compositions given in table 1 were prepared by argon arc melting, drop cast into 10 mm diameter rods, and homogeneized for 6 h at 1200 °C and 48 h at 1150 °C in vacuum at the Materials Preparation Center of Ames Laboratory^3^ 3Materials Preparation Center, Ames Laboratory USDOE, Ames, IA, USA. Specimens approximately 1 mm thick were vacuum-encapsulated in quartz capsules, further homogenized 48 h at 1150 °C in a tube furnace, and slowly brought to equilibration temperature. Equilibration treatments were conducted at 900 °C, 1100 °C and 1200 °C for 525, 100 and 50 h, respectively, followed by water quenching to retain the equilibrium microstructures. Phase constitutions were studied by x-ray diffraction (XRD) with a PANalytical Empyrean instrument, using a Co radiation source (K*α*_1_ = 1.789 Å).

**Table 1.** Nominal compositions (at.%) of the NiCoCrAlY alloys used for experimental study. All alloys contained an additional 0.1 at.% Y.

#	Al	Cr	Co	Ni
A1	24	15	19	42
A2	26	20	18	36
A3	18	28	18	36
A4	12	30	30	28
A5	16	33	30	21
A6	24	10	19	47
A7	14	16	26	44

Polished sections of the heat-treated alloys were prepared by standard metallographic procedures. Phase compositions were determined by electron probe micro-analysis (EPMA) using a JEOL JXA-8530F field emission gun instrument. For each element, measured intensities were converted to concentrations by interpolation via a calibration curve built using a series of standards of known compositions (chemical analysis by inductively coupled plasma mass spectrometry). The probe size used during measurements was about 1 *μ*m, and the alloy microstructures were sufficiently coarse for each phase to be analyzed individually.

## 3. Calphad thermodynamic models

The Al–Co–Cr, Al–Co–Ni and Co–Cr–Ni ternary systems re-modeled in [23] were combined with the Al–Cr–Ni description by Dupin *et al* [24] to produce a quaternary Al–Co–Cr–Ni extrapolation. Discrepancies in higher order systems often stem from inappropriate parameter choices in lower order systems [25]. Indeed, two distinct sets of optimized parameters may appear to describe a system equally well, but give different results when extrapolated to a higher order system. Therefore the ternary models of [23] were developed using both thermochemical data predicted from first-principles calculations [26] and quaternary experimental data from the present paper, i.e., the optimized ternary parameters were systematically extrapolated to the quaternary system to ensure consistency. This was done using the PARROT module [27] within Thermo-Calc [28, 29].

**Figure 1.**
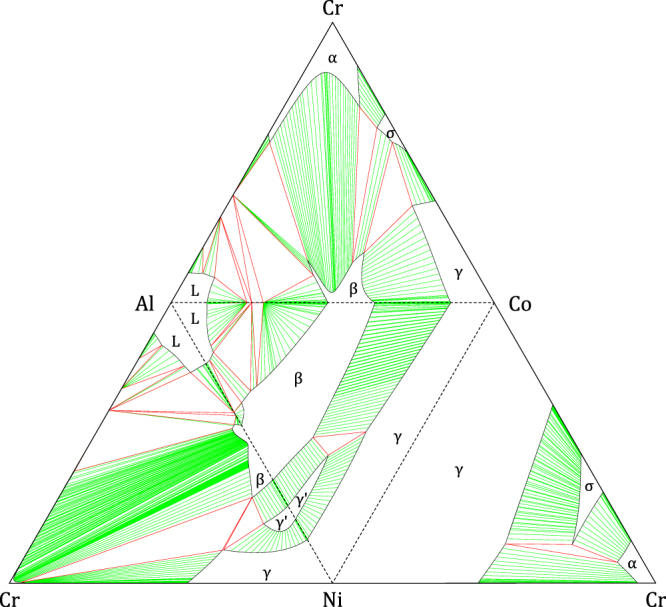
Isothermal sections (1000 °C) of the four ternary subsystems [23] used in the present work, combined to represent the four faces of the isothermal Al–Co–Cr–Ni quaternary tetrahedron.

Isothermal sections of the four ternary subsystems (1000 °C) are combined in figure 1 to represent the four faces of the isothermal quaternary tetrahedron. To extrapolate *γ*′ from Al–Cr–Ni and Al–Co–Ni into the quaternary system, interaction parameters in the quaternary system based on bond energies *U* for *γ*′-L1_2_ were introduced: 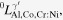 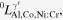 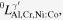 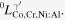 Details on the implementation of such parameters are given in [30, 31]. A quaternary model first obtained on this basis reproduced experimental phase stabilities and compositions well. However, calculated *σ* compositions had an excess of Co and Cr at the expense of Al and Ni, especially at 900 °C. Given the good agreement seen in the ternary systems containing *σ*, Al–Co–Cr and Co–Cr–Ni [23], the discrepancy was attributed to the lack of a metastable *σ* in the adopted Al–Cr–Ni model. However, alteration of binary and ternary parameters in Al–Cr–Ni for *σ* would make this phase stable, which is in fact not observed in either Cr–Ni or Al–Cr–Ni [24, 32]. A reciprocal interaction parameter (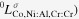 was therefore introduced to yield greater Al and Ni contents in *σ* without stabilizing it in Al–Cr–Ni. An additional metastable binary *β* interaction parameter (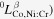 in the Co–Cr–Ni ternary was included to improve phase compositions for Co and Cr in *β*. Due to the metastability of *γ*′ in ternaries containing Co–Cr (Al–Co–Cr and Co–Cr–Ni), the ordering contribution from Co–Cr, *U*_CoCr_, was optimized using quaternary data where *γ*′ is stable, and DFT results from [23]. With these two excess parameters and one ordering parameter, a satisfactory description of the quaternary system was obtained. The PARROT module within Thermo-Calc was used to assess these parameters using phase equilibrium data from the present paper, with additional data from [19, 33] for the *γ*–*γ*′ equilibrium.

## 4. Results and discussion

### 4.1. Experimental results

**Figure 2.**
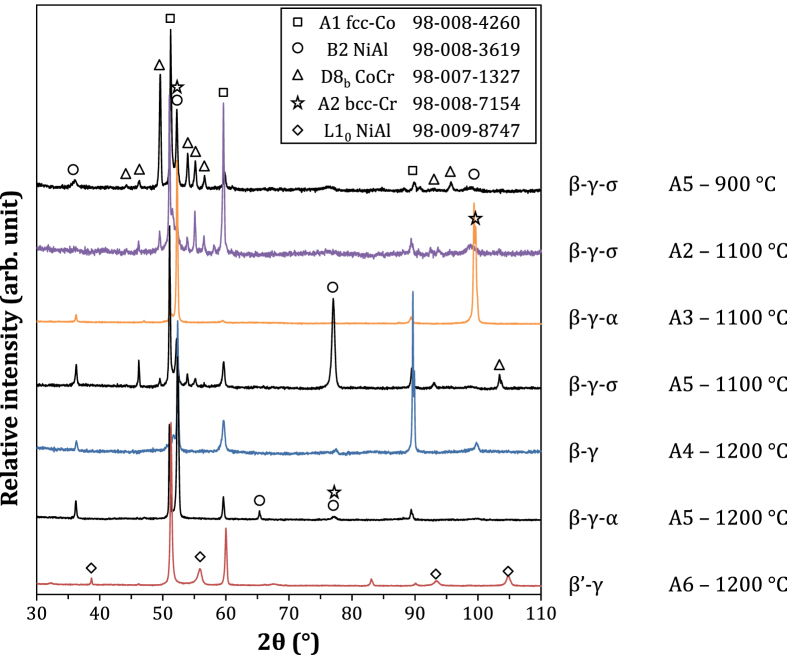
XRD analysis of phase constitution of selected alloys. The insert indicates ICSD card numbers.

**Figure 3.**
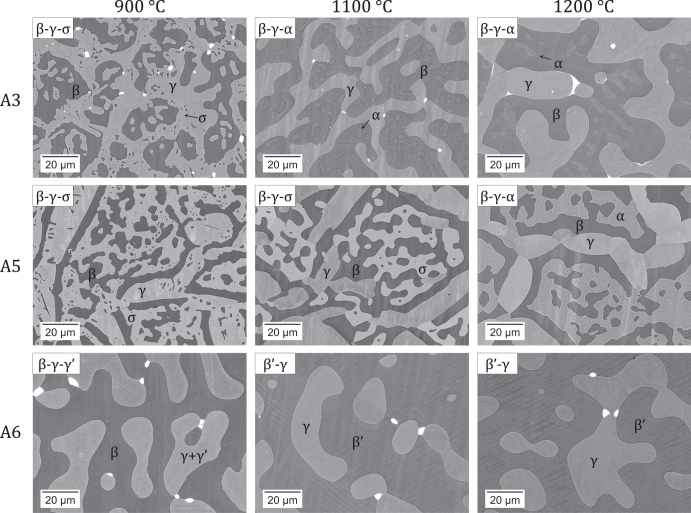
Microstructure of alloys A3, A5 and A6 equilibrated at 900 °C, 1100 °C and 1200 °C. Bright precipitates are yttrium-rich compounds.

Alloy phase constitutions were determined through a combination of XRD (selected spectra shown in figure 2), phase composition analysis (EPMA results given in table 2), and examination of the microstructures (selected micrographs shown in figure 3). In figure 3, the dark matrix is *β*, the gray phases are *α*, *γ* and *σ* (by increasing brightness), and the bright precipitates are Y-containing intermetallics. All alloys exhibit a primary *β*–*γ* microstructure typical of MCrAlY materials.

**Table 2.** Phase compositions of the NiCoCrAlY alloys measured by EPMA (at.%). Yttride compositions are not included. *γ*′ was formed in A6 and A7 at 900 °C but was too finely dispersed to be analyzed. Alloy A5 at 1100 °C was mainly *β*–*γ*–*σ*, but *σ* was locally replaced by *α* (see text); both compositions are included here.

T (°C)	#	*β*				*γ*				*σ*				*α*			
		Al	Cr	Co	Ni	Al	Cr	Co	Ni	Al	Cr	Co	Ni	Al	Cr	Co	Ni
900	A1	35.1	5.1	11.2	48.5	7.2	30.5	29.2	33.2								
	A2	36.1	6.0	12.8	45.1	6.1	34.2	32.3	27.5	1.4	59.7	28.2	10.8				
	A3	34.9	6.2	9.1	49.9	7.0	32.7	25.2	35.1	1.2	61.2	23.4	14.2				
	A4	35.9	6.5	14.0	43.6	6.1	33.5	34.0	26.3	1.4	59.0	28.9	10.7				
	A5	36.6	7.0	19.0	37.5	5.9	33.7	40.5	20.0	1.7	57.8	32.6	7.9				
	A6	33.6	3.4	11.6	51.4	9.8	20.3	29.6	40.3								
	A7	33.4	3.9	11.6	51.0	9.8	20.6	29.4	40.2								
1100	A1	32.9	8.1	13.9	45.1	9.8	28.0	27.3	34.9								
	A2	33.6	11.4	14.6	40.4	8.3	36.4	27.2	28.1	3.3	58.2	24.5	13.9				
	A3	33.0	11.6	11.5	43.9	8.7	35.9	22.4	33.0					4.6	62.1	17.8	15.5
	A4	32.7	9.8	17.7	39.7	9.2	31.0	31.9	27.9								
	A5	32.7	13.0	22.5	31.8	8.2	36.5	35.7	19.6	3.6	56.9	29.8	9.7	7.0	53.5	28.0	11.4
	A6	31.2	5.1	13.9	49.9	12.8	17.3	26.2	43.8								
	A7	31.2	5.5	14.0	49.4	12.8	17.5	26.2	43.5								
1200	A1	30.4	9.7	15.0	44.9	11.7	26.0	25.2	37.1								
	A3	29.4	15.7	13.1	41.9	10.1	35.7	21.0	33.2					9.0	49.3	18.6	23.1
	A4	30.5	12.2	19.6	37.7	10.8	29.6	30.9	28.7								
	A5	27.7	19.1	24.8	28.4	9.8	35.1	34.1	21.0					11.3	43.0	29.5	16.2
	A6	29.9	6.0	15.0	49.1	14.0	16.3	25.0	44.8								
	A7	30.0	6.6	15.3	48.2	13.8	16.8	25.4	44.0								

At 1200 °C, alloys with high chromium contents (A3, A5) produce *α* in addition to *β* and *γ*. The *α* and *β* phases both have a cubic structure, and their XRD signals could not be resolved with the instrument available. However, the phases exhibit very distinct compositions (table 2). In Cr-lean alloys (A6, A7), NiAl martensite, noted *β*′, is found instead of *β*. Martensite is identified by XRD from its L1_0_ tetragonal structure (see alloy A6 in figure 2), and presents a typical [34] twinned microstructure (figure 3). Its presence does not affect phase equilibrium since it is formed by a diffusionless transformation during cooling. At 1100 °C, the Cr-rich phase is either *α* or *σ*, depending on alloy composition. The two phases differ in their composition, with *σ* dissolving more Co and slightly less Al than *α*. Further, XRD clearly identifies the D8_b_ tetragonal structure of *σ* (see alloys A2 and A5 versus A3 in figure 2). The *σ* phase is stabilized at the expense of *α* in alloys with higher Co (A4, A5 versus A3) or lower Cr contents (A2 versus A3). In the case of alloy A5, the *σ* phase is locally replaced by *α* (i.e., there existed regions with a *β*–*γ*–*α* equilibrium); this is due to the fact that the *σ* → *α* transition temperature is very close to 1100 °C, as discussed in section 4.2. The *β* → *β*′ transformation is observed for Cr-lean alloys equilibrated at 1100 °C, but not at 900 °C (see alloy A6 in figure 3). At 900 °C, the Cr-rich alloys precipitate *σ*, and no *α* is found. Precipitation of *γ*′ in *γ* is observed for the Cr-lean alloys (A6, A7). The presence of *γ*′ could not be confirmed by XRD because its diffraction pattern was not resolved from that of *γ* with the instrument available, and *γ*′ precipitates were too small for their composition to be measured accurately by EPMA. Qualitatively, the precipitates were found to have less Cr and more Al than the matrix, and the cuboidal microstructure is typical of *γ*–*γ*′ equilibria.

If yttrium is disregarded and we consider the quaternary Al–Co–Cr–Ni system, three-phase equilibria are univariant, which explains the slight variations of the *γ*–*β*–*α* or *γ*–*β*–*σ* equilibria in the different alloys. At 1200 °C, the difference between the compositions of *α* and *β* reflects a significant miscibility gap. This is to be compared with our previous work [35], which showed that in the Al–Co–Cr system the *α*–*β* (A2–B2) miscibility gap was suppressed and replaced by an order–disorder transition between 1100 °C and 1200 °C. In contrast, Dupin *et al* [24] showed that the mutual solubilities of *α* and *β* remained very limited up to melting in the NiAl–Cr pseudo-binary system. Thus in terms of the *α*–*β* phase relationship, the Al–Co–Cr–Ni system exhibits an intermediate behavior between those of Al–Co–Cr and Al–Cr–Ni. The tendency to a disordering of the *β* structure with increasing temperature is observed here as the Al and Cr contents of the *β* phase show large variations at 1200 °C (table 2). Higher Co contents favor an increase of Cr solubility and decrease of Al content in *β*, as could be expected from the behavior of the Al–Cr–Ni and Al–Co–Cr systems. As the temperature decreases to 1100 and 900 °C, the range of Al and Cr concentrations in *β* is restricted.

In terms of alloy microstructures, *α* precipitation mainly occurred within *β*, as expected considering the similar crystal structure of these two cubic phases. Precipitation of *σ* is seen in figure 3 to have occurred both from *α*, reflecting the replacement of *α* by *σ* in the three-phase equilibrium as the temperature decreases, and at the expense of *γ* at the *β*–*γ* interface, driven by the decrease of Cr solubility in *γ*. In the latter case, heterogeneous nucleation reflects the lack of compatibility between the tetragonal *σ* and the other phases of the system. In contrast, *γ*′ is exclusively found within *γ*. Coherent precipitation is typically observed for these two cubic phases.

### 4.2. Calphad modeling results

**Figure 4.**
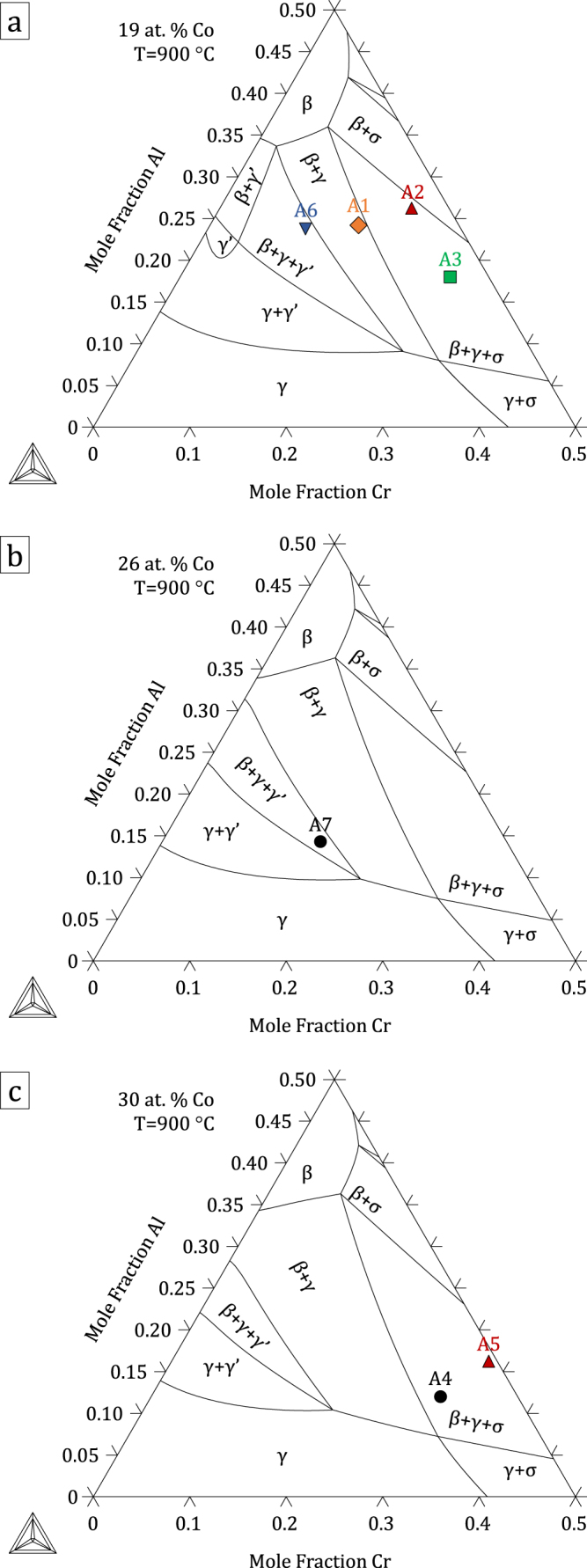
Isothermal (900 °C), isoplethal Al–Co–Cr–Ni sections at (a) 19, (b) 26 and (c) 30 at.% Co. Nominal compositions of the alloys used in this study are indicated. Note that some alloys shown in (a) are not strictly in the plane of the calculation as their Co contents are slightly different from 19 at.%.

**Figure 5.**
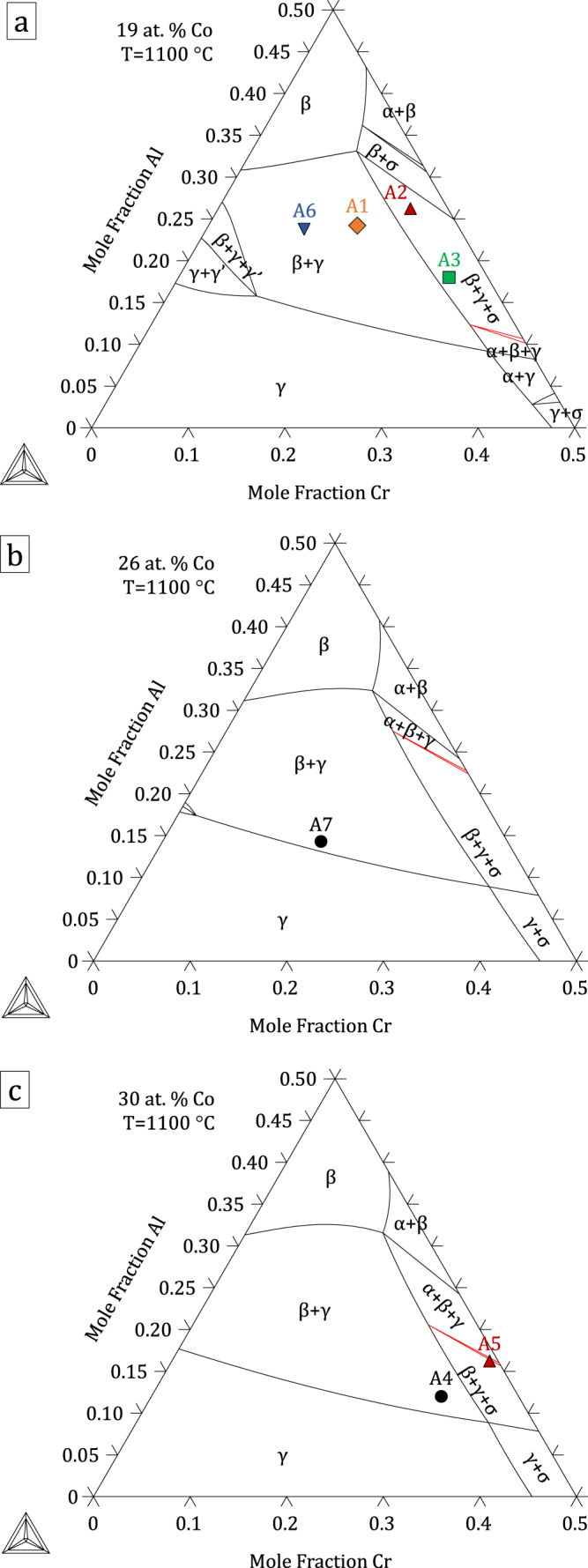
Isothermal (1100 °C), isoplethal Al–Co–Cr–Ni sections at (a) 19, (b) 26 and (c) 30 at.% Co. Nominal compositions of the alloys used in this study are indicated. Note that some alloys shown in (a) are not strictly in the plane of the calculation as their Co contents are slightly different from 19 at.%.

**Figure 6.**
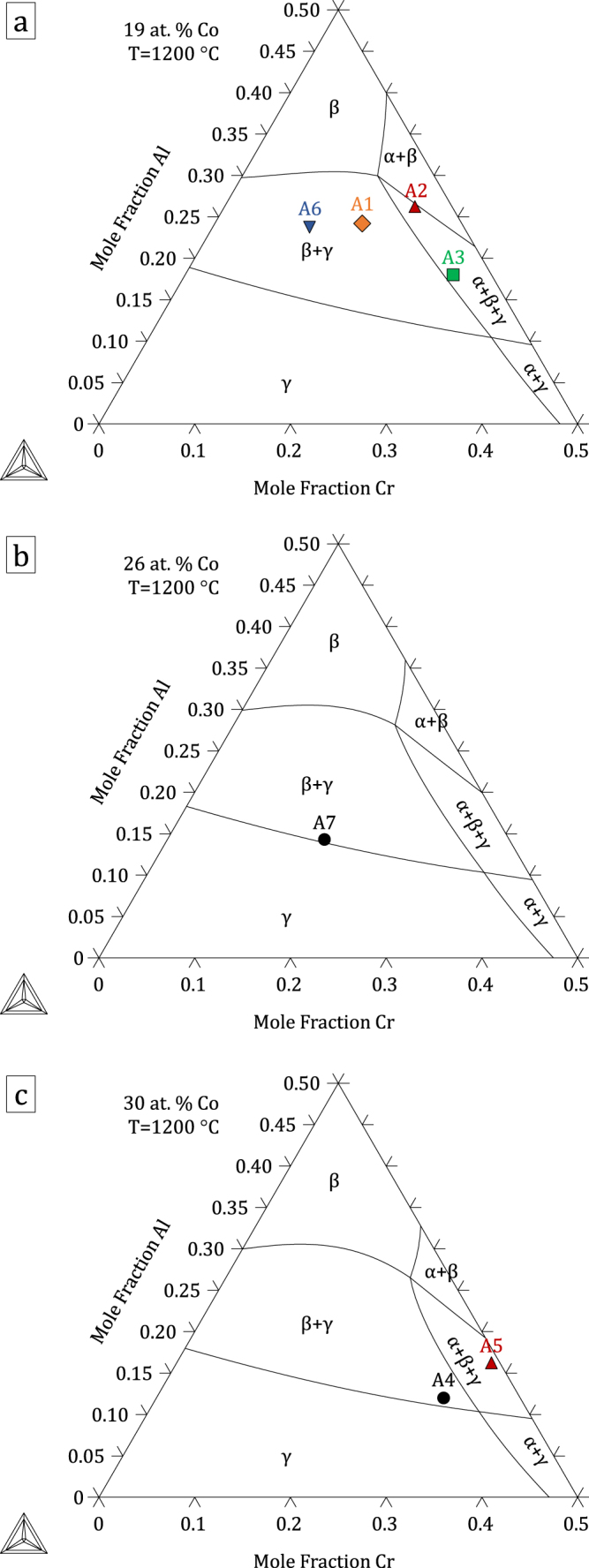
Isothermal (1200 °C), isoplethal Al–Co–Cr–Ni sections at (a) 19, (b) 26 and (c) 30 at.% Co. Nominal compositions of the alloys used in this study are indicated. Note that some alloys shown in (a) are not strictly in the plane of the calculation as their Co contents are slightly different from 19 at.%.

**Figure 7.**
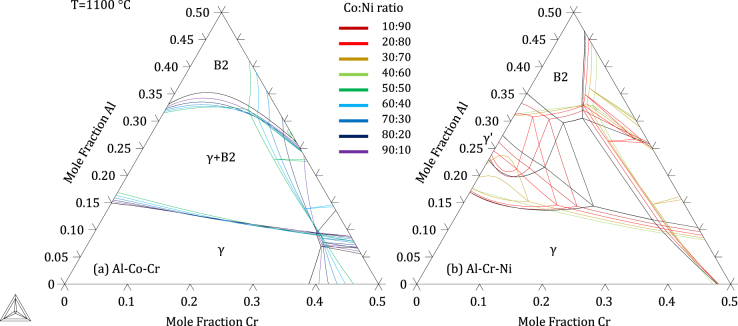
Isothermal (1100 °C) sections of the Al–Co–Cr–Ni system plotted for constant Co:Ni ratios (see color scale inserted). Cobalt-rich and Ni-rich sections are superimposed on the (a) Al–Co–Cr and (b) Al–Cr–Ni diagram, respectively. A combination of the two plots describes the full quaternary system as a function of the Co:Ni ratio.

The thermodynamic parameters resulting from the present assessment, given in the supplementary material: File S1, were built into a database included in File S2. Figures 4–6 show calculated quaternary isoplethal, isothermal sections at three cobalt contents (19, 26, and 30 at.%) and temperatures (900 °C, 1100 °C, and 1200 °C) with nominal alloy compositions overlaid. Correct phase constitutions are predicted for all alloys based on their nominal compositions, except in one case: at 1100 °C, alloy A3 is predicted to be in a *β*–*γ*–*σ* field, while it was found to form *α* and not *σ* (figure 2). For this particular composition, the *α* → *σ* transition temperature is calculated to be 1109 °C, quite close to 1100 °C. In the case of alloys A6 and A7 at 900 °C, *γ*′ was predicted to form, in agreement with our observations (see A6 in figure 3). Figure 7 shows isothermal (1100 °C) sections at constant Co:Ni ratios from 10:90 to 90:10, superimposed on the Al–Co–Cr and Al–Cr–Ni diagrams. The destabilizing effect of cobalt on *γ*′ is clearly illustrated in figure 7(b), where the *γ*′ region is seen to shrink with increasing Co:Ni ratio.

**Figure 8.**
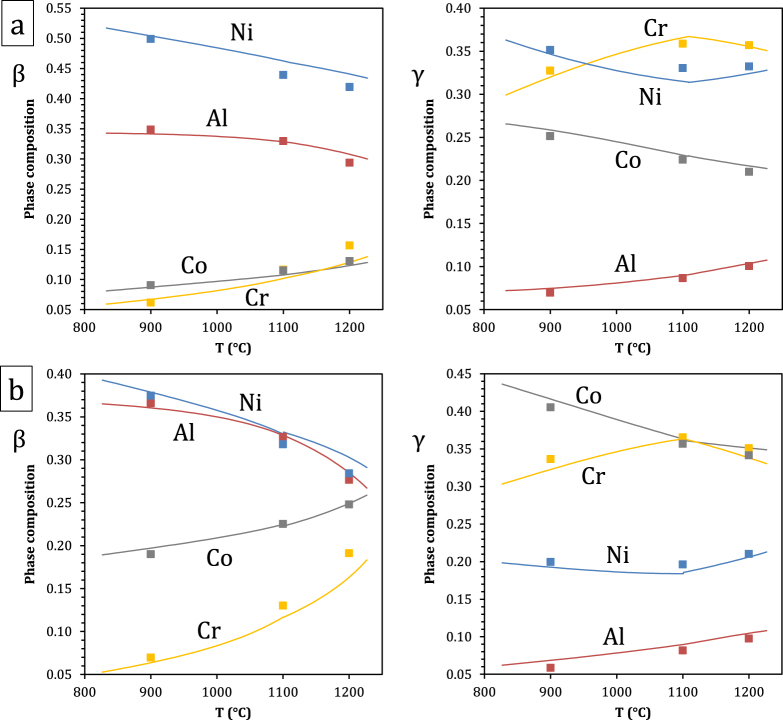
Composition of the *β* and *γ* phases of alloys (a) A3 and (b) A5, calculated and measured by EPMA.

**Figure 9.**
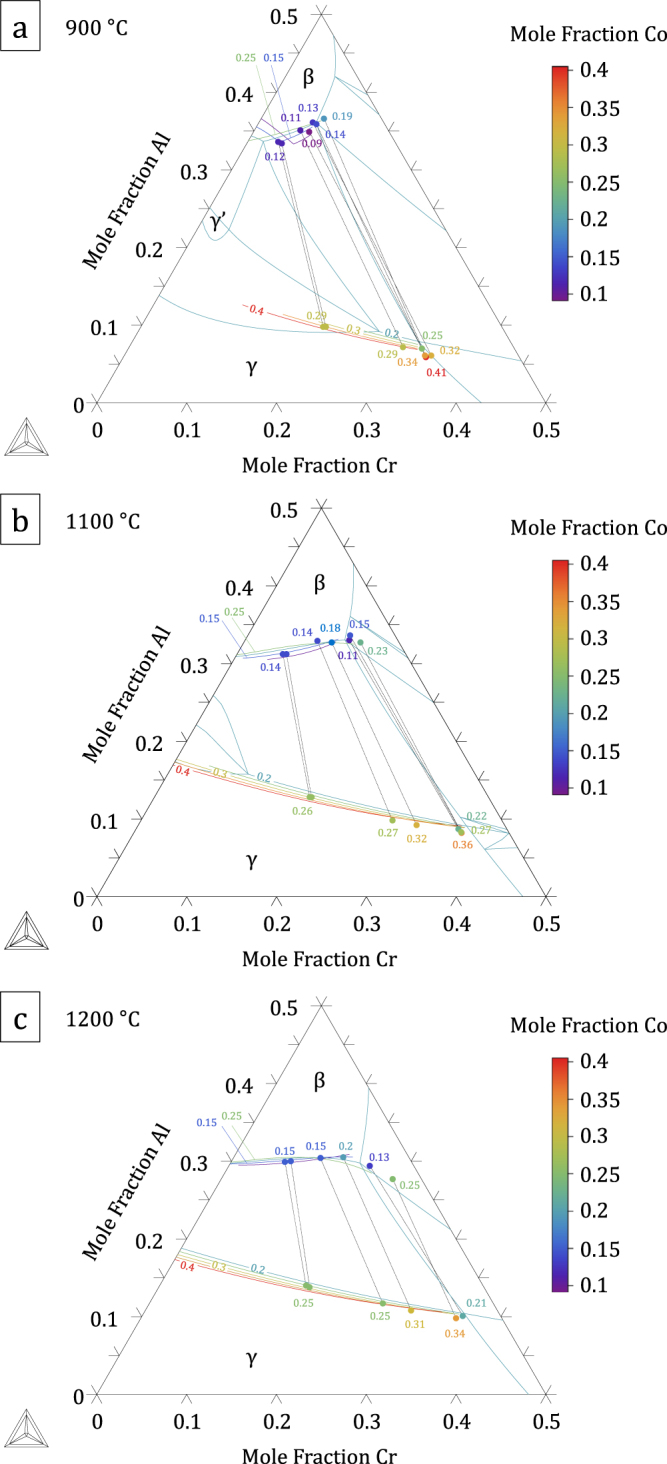
Isothermal, Co-isoplethal sections of the Al–Co–Cr–Ni system at (a) 900 °C, (b) 1100 °C and (c) 1200 °C. The *β*/*β* + *γ* and *β* + *γ*/*γ* phase boundaries are represented for various Co contents using the indicated color code, forming a Co contour map. Experimental *β* and *γ* compositions are given, where the same color code has been applied to the end points, and measured Co concentrations are also indicated. Tie-lines projected on a plane of constant Co are also added to indicate the compositions in equilibrium.

**Figure 10.**
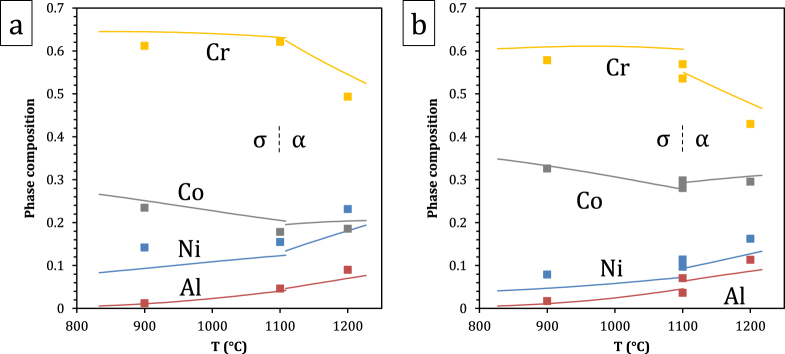
Composition of the *σ* and *α* phases of alloys (a) A3 and (b) A5, calculated and measured by EPMA.

A comparison of predicted and measured phase compositions is provided in figures 8–10. The calculated *β*–*γ* equilibria are in very good agreement with the experimental data. This is illustrated in figure 8 where phase compositions calculated on the basis of nominal alloy compositions are plotted as a function of temperature for alloys A3 and A5, together with experimental data. In figure 9, isothermal *β*/*β* + *γ* and *β* + *γ*/*γ* phase boundaries from isopleths are plotted with colors corresponding to constant cobalt contents, thus forming a contour map. Experimental *β*–*γ* tie-lines are superimposed, where the same color code has been applied to the end-points, and measured *x*_Co_ values are also indicated. All alloys are represented, but the *α* and *σ* phases were omitted. The database is evaluated by comparing the color (i.e., cobalt content) of a datapoint with that of the surrounding phase boundaries. Again a very good agreement is observed: even though the Al and Cr solubilities in *β* and *γ* show relatively limited variations with cobalt content in the compositional space studied, the trends observed experimentally are well reproduced by the calculation.

In the case of *σ* and *α*, the agreement is slightly less good, as shown in figure 10, where phase compositions are plotted as a function of temperature for alloys A3 and A5. The *σ* → *α* transition temperature for alloy A5 is calculated to be 1101 °C. Experimentally, both regions of *β*–*γ*–*σ* and of *β*–*γ*–*α* equilibrium were observed at 1100 °C. Accuracy in the annealing temperature or the calculated transition temperature is not expected to be better than one degree; whether the actual temperature was slightly above or slightly below the transition temperature, equilibrium over macroscopic distances would require remarkable compositional uniformity, and excessively long annealing time: here small heterogeneities locally stabilize either *σ* or *α*. This is also illustrated in figure 5(c), where the nominal composition of alloy A5 is seen to be close to the *β*–*γ*–*σ*–*α* and *β*–*γ*–*α* fields at this temperature. Both *σ* and *α* compositions are plotted in figure 10, and the database is seen to predict the trend observed upon the phase transformation reasonably well. The small discrepancy can be attributed to the sublattice model implemented here for *σ*, which does not offer the best description of this CoCr-base *σ*, as discussed in detail by Joubert [36]. In the current model, Cr can take on a maximum composition of 73.3 at.% at the end-member (A)_8_(Cr)_18_(Cr)_4_ (where A is Al or Co). A non-simplified *σ* model would include five distinct sublattices relating to Wyckoff positions with all four Al, Co, Cr, and Ni allowed to occupy each sublattice: (Al, Co, Cr, Ni)_2_(Al, Co, Cr, Ni)_4_(Al, Co, Cr, Ni)_8_(Al, Co, Cr, Ni)_8_(Al, Co, Cr, Ni)_8_ [36, 37]. Within this model, 1024 end-members would have to be defined. Simplifications made in [23, 35] by combining certain sites and restricting site occupation, which are commonly made [36], were thus retained here. A model based on a better suited site combination exists [36], which would lead to a maximum Cr concentration of 66.7 at.% at the end-member (A)_10_(Cr)_16_(Cr)_4_. With similar site occupations, this would yield Cr concentrations closer to experimental results. Changing sublattice models would require remodeling the Co–Cr binary and associated ternaries; this was outside the scope of the current work but will be investigated in the future. The resulting discrepancy is limited (1–5 at.%) and the compositional trends are respected, such that the rest of the system is little affected. Overall, the present quaternary modeling provides a very good description of all phase compositions.

### 4.3. Comparison with existing databases

**Figure 11.**
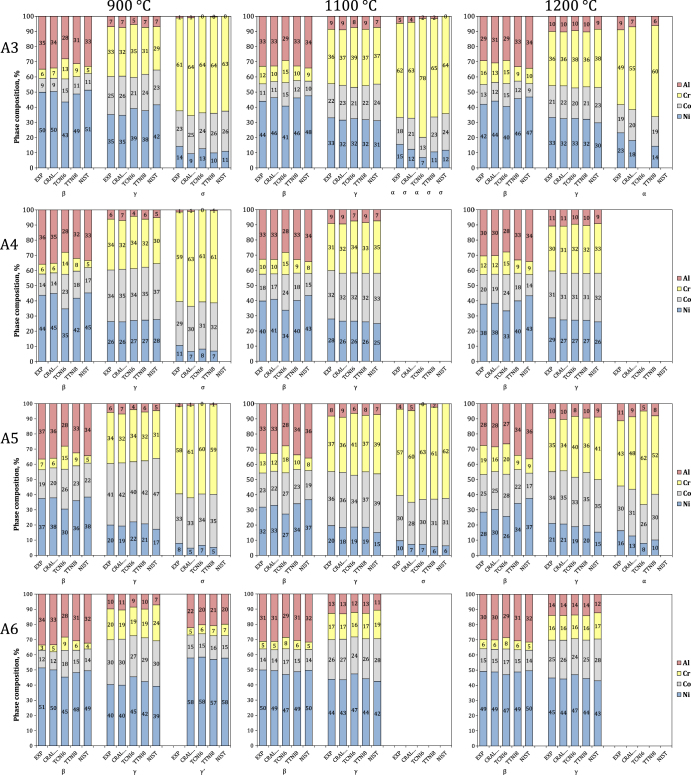
Comparison of experimental (EXP) and calculated phase compositions for alloys A3, A4, A5 and A6 at 900 °C, 1100 °C and 1200 °C. Calculations based on the present developmental database (CRALDAD), and existing databases (TCNI6, TTNI8, NIST).

Figure 11 shows phase compositions calculated on the basis of nominal alloy compositions using our developmental database (CRALDAD1 for chromium and aluminium DFT-augmented database 1), and three existing databases (TCNI6 [38], TTNI8 [39], NIST Ni-base superalloy [40]), compared with the present experimental results. Four alloys (A3, A4, A5, A6) were selected so as to cover a large compositional region of the system. All calculations were conducted using Thermo-Calc, with the same conditions. The newer TCNI7 was later verified to produce the same results as the TCNI6 results included here.

As a general observation, CRALDAD offers the best fit to the experimental results. This could be expected since it was built using these results. The important criterion in evaluating a database is its consistency over a large range of temperatures and compositions, ideally assessed with a number of independent experimental datasets. Reliable experimental data in the quaternary system are lacking, thus in the following the four databases are simply evaluated against the present dataset.

The CRALDAD, TCNI6 and TTNI8 databases provide a relatively good depiction of the *γ* phase, while that given by NIST presents a number of issues. For example, the solubility of Cr in *γ* is overestimated at 1200 °C and 1100 °C, especially for Co-rich alloys (A4, A5), but underestimated at 900 °C. Inconsistencies are also noted in the Ni/Co ratio in *γ*: NIST tends to overestimate Co and underestimate Ni at 1200 °C and 1100 °C and in the case of alloy A5 at 900 °C, but at the lower temperature, the contrary is observed for the Co-lean alloy A3. Only CRALDAD and TTNI8 provide a very accurate representation of Al solubility in *γ* at all three temperatures, although the variance obtained with TCNI6 is small (<2 at.% difference compared to the experimental data).

Modeling of the *β* phase is subject to more discrepancy. Only CRALDAD provides a consistently good description, while the quality of that obtained with the other databases shows significant variations with temperature. TCNI6, for instance, offers a relatively good fit at 1200 °C, but proves inadequate at 1100 °C and 900 °C, as it underestimates Al and Ni, and overestimates Cr and Co, with rather large errors (up to 9 at.% versus the experimental data). On the contrary, the compositions calculated from NIST show large differences with experimental data at 1200 °C (up to 10 at.%), but the spread is reduced at the lower temperatures. Finally, TTNI8 is at significant variance at 1200 and 900 °C, but proves excellent at 1100 °C.

Significant discrepancies also emerge in the case of the Cr-rich phase, which is either *α* or *σ* depending on temperature and alloy composition. The NIST database fails to account for *α* formation at 1200 °C, predicts *σ* instead of *α* in alloy A3 at 1100 °C, and does not always predict *σ* formation at 900 °C, depending on alloy composition. Furthermore, it does not account for any Al solubility in *σ* when it successfully predicts its formation. CRALDAD offers the best representation of *α* at 1200 °C: it overestimates Cr and underestimates Ni and Al, but less so than TTNI8 and TCNI6. All databases tend to overestimate Cr, and underestimate Ni, in *σ*. CRALDAD provides the best description of *σ* at 1100 °C—in particular, it accounts for the relatively high Al solubility–but it tends to overestimate the Cr content slightly more than TTNI8 or TCNI6 do at 900 °C. As mentioned earlier, the *σ* model implemented in CRALDAD is not the best adapted. TCNI6 uses a model better suited to CoCr-base *σ*, which should produce a lower Cr content; however, the composition given by TCNI6 is only slightly better than that of CRALDAD at 900 °C, and TCNI6 actually yields a higher Cr content at 1100 °C. The absence of a clear advantage for TCNI6 illustrates the complexity of modeling a quaternary system.

The descriptions of *γ*′ could not be evaluated due to the absence of experimental data. Compositions calculated for alloy A6 at 900 °C are all similar. More experimental input is required to correctly assess this part of the quaternary system, but the fact that CRALDAD successfully predicted *γ*′ formation is encouraging, since it was built more specifically toward the description of the equilibrium of *β* and *γ* with either *α* or *σ*.

The NIST, TCNI6 and TTNI8 databases were built with the aim of modeling nickel base superalloys, which have a *γ*–*γ*′ microstructure and significantly less Al, Cr and Co than the NiCoCrAlY alloys considered here. While the compositions of phases in equilibrium are evidently related via a mass balance, this rationalizes the fact that all three databases provided better representations of *γ* than they did for the Al-rich *β* or the Cr-rich *α* and *σ*. This also explains why these databases best represented the *β*–*γ* equilibrium in alloy A6, which has less Cr than the other alloys. In the case of the NIST database, the Al–Co–Cr subsystem is not assessed, and *σ* is modeled as a binary CoCr compound, with no Al solubility. This in turns affects the composition of the *γ* and *β* phases, and partly explains the discrepancies observed here between experimental and calculated compositions. The Al solubility in *σ* also tends to be underestimated by TCNI6. Despite the *σ* model used in CRALDAD being less well adapted than that of TCNI6, the parameter optimization in CRALDAD allows the high Al solubility to be accounted for. Outside of the *σ* phase, the good consistency of CRALDAD over the 900 °C–1200 °C temperature range reflects the effort to include, as much as possible, physically correct descriptions of all phases in the underlying ternary subsystems.

## 5. Conclusions

Extrapolation of the re-assessed ternary subsystems into the Al–Co–Cr–Ni system led to a description in very good agreement with experimental data, using a minimum number of quaternary interaction parameters. This assessment was based on a set of EPMA and XRD data obtained over a relatively wide range of compositions and temperatures. Primarily designed toward MCrAlY coatings, our database allows excellent representation of phase boundaries in *β*–*γ* equilibria at 900 °C–1200 °C, and correctly accounts for three-phase *β*–*γ*–*σ* and *β*–*γ*–*α* equilibria in alloys rich and Co and Cr. The *σ* → *α* transition temperature, as well as compositional trends in the Cr-rich phases, are well described. Leads for an improved description of *σ* are identified. Precipitation of *γ*′ at 900 °C in Cr-lean alloys was successfully predicted, although a finer assessment will require more experimental data. Comparison with existing databases on the basis of a set of measured phase compositions is favorable to our developmental database.
